# Oocytes With Smooth Endoplasmic Reticulum Aggregates May Not Impact Blastocyst Euploidy Rate

**DOI:** 10.3389/fendo.2022.851370

**Published:** 2022-08-24

**Authors:** Jian Xu, Li Yang, Zhi-Heng Chen, Min-Na Yin, Juan Chen, Ling Sun

**Affiliations:** Center of Reproductive Medicine, Guangzhou Women and Children’s Medical Center, Guangzhou Medical University, Guangzhou, China

**Keywords:** smooth endoplasmic reticulum aggregates, PGT, oocyte, blastocyst, euploidy rate

## Abstract

**Objective:**

To investigate whether the euploidy rate of blastocysts derived from smooth endoplasmic reticulum aggregates (SERa) positive cycles and oocytes are impacted.

**Design:**

Retrospective cohort study.

**Method(s):**

A total of 601 preimplantation genetic testing (PGT) cycles with at least one oocyte retrieved in our center between April 2017 and May 2021 were initially included in the study. Women>35 years and PGT cycles with chromosomal structural rearrangements (PGT-SR) were excluded. Embryological and blastocyst ploidy outcomes were compared among SERa+ oocyte, sibling SERa- oocytes and oocytes in SERa- cycles.

**Results:**

No significant difference was observed among the SERa+ oocyte group, sibling SERa- oocyte group, and SERa- cycle group in the normal fertilization rate (82.1% vs. 77.8% vs. 83.1%, respectively, *P*=0.061), blastocyst formation rate (71.0% vs. 72.5% vs. 68.4%, respectively, *P*=0.393), good quality blastocyst formation rate (46.4% vs. 48.3% vs. 42.6%, respectively, *P*=0.198). No significant difference was observed in the euploidy rate (50.0% vs. 62.5% vs. 63.3%, respectively, *P*=0.324), mosaic rate (12.5% vs. 9.7% vs. 13.4%, respectively, *P*=0.506), and aneuploidy rate (37.5% vs. 27.8% vs. 23.2%, respectively, *P*=0.137) among the three groups.

**Conclusion:**

Our results suggest that the euploidy rate of blastocysts derived from SERa+ cycles and oocytes may not be impacted.

## Introduction

Aggregates of smooth endoplasmic reticulum (SERa) in the ooplasm are one of the cytoplasmic dysmorphisms of oocytes. These aggregations appear as round flat disks in the ooplasm corresponding to large tubular SER clusters surrounded by mitochondria (1). The occurrence rates of SERa reported in papers are different, ranging from 5.4% to 23.1% (2).

Significantly lower pregnancy rates were first reported in 2004 and a baby diagnosed with Beckwith-Wiedemann syndrome was born in SERa+ cycles (3). Since then, several studies have shown significantly reduced pregnancy rates and a comparatively high number of congenital abnormalities in live-born babies derived from SERa+ oocytes and/or cycles (1, 4–6). Due to these adverse outcomes, in 2011 the Istanbul consensus recommended not to use SERa+ oocytes (7).

However, following this recommendation, no increase in congenital anomalies in embryos derived from SERa+ oocytes was observed in other studies, and there did not seem to be reduced pregnancy rates (8–10). It was reported that only 14% of centers discarded SERa+ oocytes (11). Consequently, the revised Vienna Alpha/ESHRE consensus reconsidered the recommendation and advised a case-by-case approach in 2017 (12).

In our recent study, 43 embryo transfer cycles from SERa- patient were matched to the 43 transferred cycles with pure SERa+ oocyte derived embryos. We found that the implantation, clinical pregnancy, and live birth rate of embryos derived from oocytes with SERa are not impaired. Twenty-eight healthy babies without any major malformations were born after transfer of embryos originating from SERa+ oocytes (13).

Aneuploidy is responsible for more than half of all missed abortions (14, 15), and it is the leading cause of congenital birth defects (16, 17). Recently, Otsuki and colleagues reported that the incidence of mitotic cleavage failure and the incidence of meiotic cleavage failure during the second polar body extrusion in oocytes with SERa were significantly higher than that in oocytes without SERa. They speculated the blastocyst originated from SERa+ oocyte may further turn into aneuploidy (2). However, to the best of our knowledge, most current studies which investigated the effect of SERa on embryos focused on its impact on fertilization and early embryo development. Only one abstract with small sample size (no specific data) mentioned that the euploidy rate of blastocysts derived from SERa+ ooctyes decreased (18).

In this article, we analyzed whether the embryos derived from SERa+ oocyte and sibling SERa- oocyte were associated with negative embryological outcomes, and more importantly, whether the euploidy rates of blastocysts derived from SERa+ oocyte, orsibling SERa- oocyte were negatively impaired in younger non-PGT-SR cycles.

## Materials and Methods

### Study Design and Study Participants

All women undergoing preimplantation genetic testing (PGT) cycles with at least one oocyte retrieved in the Centre for Reproductive Medicine, Guangzhou Women and Children’s Hospital between April 2017 and May 2021 were included in this retrospective cohort study. Because embryo aneuploidy rates increase significantly after age 35 (19), women>35 years were excluded to avoid the confounding factor of advanced maternal age. PGT cycles with chromosomal structural rearrangements (PGT-SR) were excluded due to the potential correlation between chromosomal structural rearrangements and decrease in embryo development and embryo euploidy rate (20). Parts of PGT cycles with monogenic defects (PGT-M) cycles without blastocyst euploidy analysis were also excluded. The study was approved by the Independent Ethics Committee of Guangzhou Women and Children’s Hospital (No. 2020-57801).

SERa+ oocytes were defined as those oocytes where one or more SERa were visible with an inverted microscope after denudation just prior to intracytoplasmic sperm injection (ICSI). A SERa+ cycle indicates that at least one SERa+ oocyte is observed among the cohort. The SERa- cycles had morphologically normal oocytes.

The eligible cycles were divided into SERa+ cycle group and SERa- cycle group. The SERa+ cycle group was further subdivided into SERa+ MII oocyte group and sibling SERa- MII oocyte group. The primary outcome measures were euploidy, mosaicism, and aneuploidy. Secondary endpoints were comparison of normal fertilization rate, blastocyst formation rates, and good quality blastocyst formation rate.

### Embryo Culture and Blastocyst Biopsy

**Figure 1:**
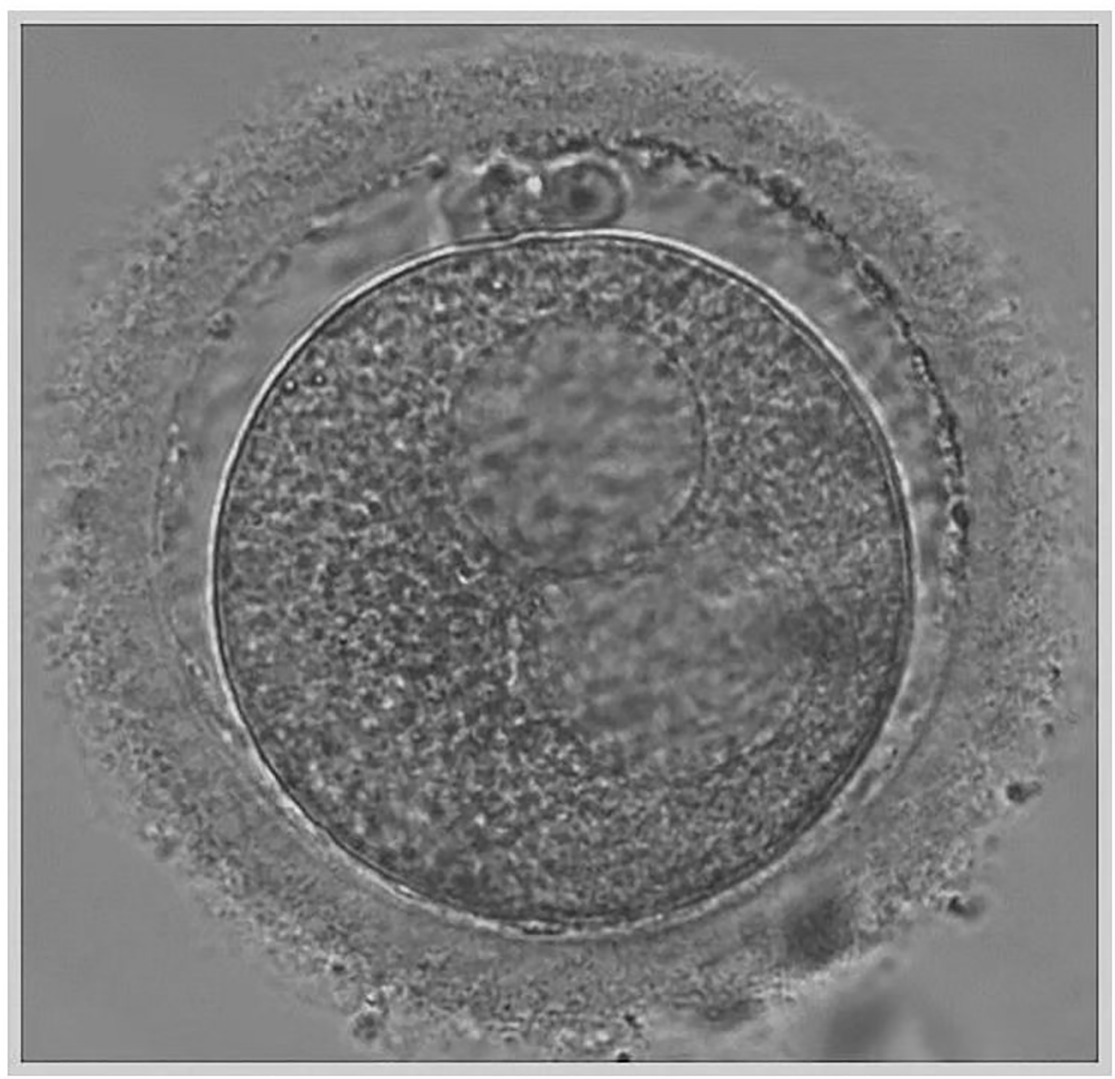
Metaphase II oocyte displaying the SER dysporphism.

Transvaginal sonographic oocyte retrieval was performed 34–36 h after trigger, and conventional ICSI was performed 4–6 h after the oocyte retrieval. At the time of ICSI, each oocyte was evaluated for the presence of cytoplasmic abnormalities using an inverted microscope and data were recorded. The large SERa present in the cytoplasm of MII resembles a vacuole but can be easily distinguished from a vacuole since it is not fluid filled and not separated from the rest of the cytoplasm by a membrane (**Figure 1**) (21). Only mature MII oocytes were injected, and the formation of SERa was found only in MII stage oocytes in our center. During the ICSI procedure, we carefully avoided rupturing the aggregate, and fertilization was checked 16–18 h after insemination. Zygotes were cultured individually in G1 (Vitrolife, Sweden) media under 6% CO_2_, 5% O_2_, 37°C until day three in humidified incubator. Embryos were then transferred on day three to G2 (Vitrolife, Sweden) media and cultured individually under the same conditions until day five or six. A good quality blastocyst was defined according to Gardner and Schoolcraft grading (22), embryos must have reached a minimum score of 3BB. All the good quality blastocysts were subjected to trophectoderm cell-biopsy by laser on day 5 or day 6 and 5–10 TE cells were biopsied. After biopsy, blastocysts were cryopreserved using vitrification according to the manufacturer’s protocol (Jieying Vitrification Kit, Jieying Laboratory Inc., Canada). Briefly, blastocysts were equilibrated in V1 solution for 5 min at room temperature, exposed to V2 solutions for 1 min at 25°C, and immediately loaded onto straw. The straw was then quickly submerged into liquid nitrogen and stored.

### NGS Protocol for the TE Biopsy

The multiple displacement amplification (MDA, Qiagen) DNA amplification system was used for whole genome amplification (WGA) to generate sufficient DNA for analysis. The amplification reactions were performed by incubating at 30°C for 8 h and then heat-inactivated at 65°C for 3 min, according to the manufacturer’s protocol (Qiagen, Germany). The Illumina MiSeq platform was used for NGS, and approximately 1.5 million fragments of amplified DNA from each TE biopsy were sequenced. An on-instrument computer performed primary and secondary data analysis to align the reads to a reference genome. PGXcloud cloud server (available at http://www.pgxcloud.com/) was used to analyze the chromosomal copy number variants (CNVs) (Jabrehoo, China). All profile reports were analyzed independently by two laboratory technicians. In the event of any differences in final assessment between the technicians, a consensus was reached after further team discussion.

Embryos with available PGT-A results were classified as euploid, mosaic, or aneuploid. Embryos with less than 20% aneuploidy in the TE sample were classified as euploid; those between 20% and 80% were reported as mosaic, while those over 80% were classified as aneuploid; this is in conformation with the current guidelines of Preimplantation Genetics Diagnosis International Society (23).

### Statistical Analysis

The quantitative variables are presented as the mean ± standard deviation. Statistical comparisons of two experimental groups were evaluated by the Mann-Whitney U test after data distribution failed the normality test. The categoric variables are expressed as frequency and percentage. A chi-square test or Fisher’s exact test were used to compare normal fertilization rate (defined as the ratio between the number of 2PN oocytes and the number of MII oocytes injected), blastocyst formation rate (defined as the ratio between total number of blastocysts formed and the number of embryos cultured up to Days 5–6), good quality blastocyst rate (defined as the number of good quality blastocysts per number of retrieved MII oocytes), and euploidy rate, etc. *P* value <0.05 was considered statistically significant.

## Results

**Figure 2:**
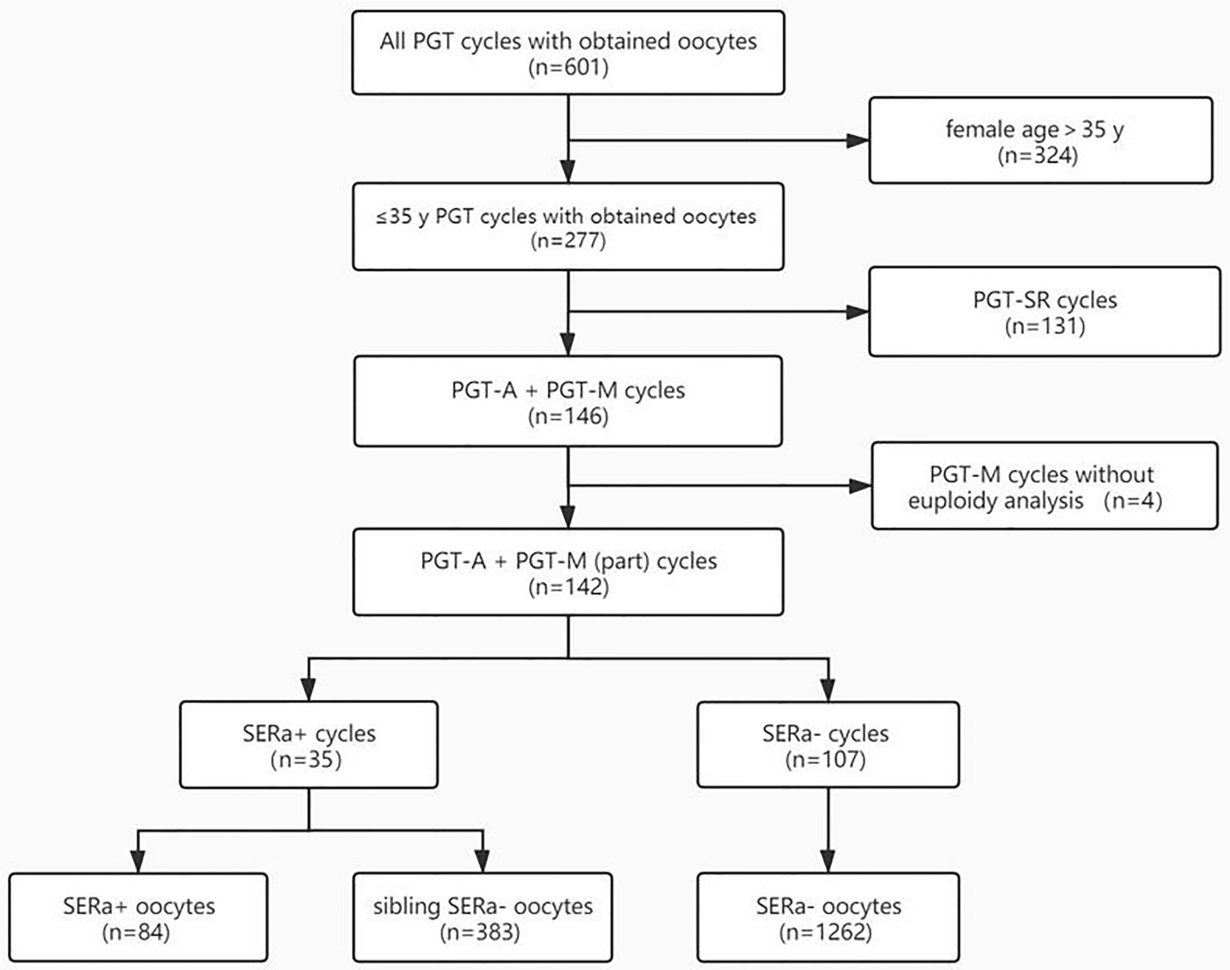
Flowchart. PGT-SR, PGT for chromosomal structural rearrangements; PGT-A, PGT for aneuploidy; PGT-M, PGT for monogenic defects; SERa, smooth endoplasmic reticulum aggregation.

A total of 601 PGT cycles with at least one oocyte retrieved were reviewed for eligibility. Among them, women more than 35 years old, PGT-SR cycles, and PGT-M cycles without blastocyst euploidy analysis were excluded from outcome analyses. Therefore, a total of 142 PGT cycles from 116 women were included in the outcome analyses (**Figure 2**).

As shown in **Table 1**, the mean age (31.1 vs. 30.5, *P*=0.249), mean number of retrieved oocytes (16.2 vs. 15.0, *P*=0.505), mean number of MII oocytes (13.4 vs. 11.8, *P*=0.264), and stimulation protocols (*P*=0.988) were comparable between the SERa+ cycles group (n=35) and SERa- cycles group (n=107).

**Table 1:** Baseline characteristics.

	SERa+ cycles (n=35) (30 patients)	SERa- cycles (n=107) (86 patients)	*P*
Age (year)	31.1 ± 3.0	30.5 ± 2.9	0.249
Mean no. of retrieved oocytes	16.2 ± 9.7	15.0 ± 8.3	0.505
Mean no. of MII oocytes	13.4 ± 8.7	11.8 ± 6.8	0.264
Stimulation protocols			0.988
antagonist protocol	21	63	
long protocol	11	34	
other protocols	3	10	
PGT type			0.688
PGT-A	14	47	
PGT-M	21	60	

SERa, smooth endoplasmic reticulum aggregation; PGT-A, preimplantation genetic testing for aneuploidy; PGT-M, preimplantation genetic testing for monogenic defects.

The SERa+ cycle group was subdivided into SERa+ MII oocyte group and sibling SERa- MII oocyte group. The main embryological outcomes were compared among SERa+ MII oocytes (n=84), sibling SERa- MII oocytes (n=383), and MII oocytes (n=1262) in the SERa- cycles in the selected PGT cycles (**Table 2**). No significant difference was observed in the normal fertilization rate (82.1% vs. 77.8% vs. 83.1%, respectively, *P*=0.061), blastocyst formation rate (71.0% vs. 72.5% vs. 68.4%, respectively, *P*=0.393), good quality blastocyst formation rate (46.4% vs. 48.3% vs. 42.6%, respectively, *P*=0.198) among the three groups.

**Table 2:** Embryological and blastocyst ploidy outcomes for SERa+ oocyte, sibling SERa- oocytes and oocytes in SERa- cycles.

	SERa+ cycles (n=35) (30 patients)		SERa- cycles(n=107) (86 patients)	*P*
	SERa+ MII oocytes	sibling SERa- MII oocytes	MII oocytes	
	n=(84)	n=(383)	n=(1262)	
Percent 2 pronuclei (2PN)/MII	69/84 (82.1%)	298/383 (77.8%)	1049/1262 (83.1%)	0.061
Blastulation (%)	49/69 (71.0%)	216/298 (72.5%)	718/1049 (68.4%)	0.393
Percent good quality blastocysts	32/69 (46.4%)	144/298 (48.3%)	447/1049 (42.6%)	0.198
Percent euploidy blastocysts	16/32 (50.0%)	90/144 (62.5%)	283/447 (63.3%)	0.324
Percent mosaic blastocysts	4/32 (12.5%)	14/144 (9.7%)	60/447 (13.4%)	0.506
Percent aneuploidy blastocysts	12/32 (37.5%)	40/144 (27.8%)	104/447 (23.2%)	0.137

SERa, smooth endoplasmic reticulum aggregation; MII, metaphase II.

The ploidy rates of formed good quality blastocysts derived from SERa+ oocytes group (n=32), sibling SERa- oocytes group (n=144), and MII oocytes in the SERa- cycles (n=447) are compared (**Table 2**). Similarly, no significant difference was observed in the euploidy rate (50.0% vs. 62.5% vs. 63.3%, respectively, *P*=0.324), mosaic rate (12.5% vs. 9.7% vs. 13.4%, respectively, *P*=0.506), and aneuploidy rate (37.5% vs. 27.8% vs. 23.2%, respectively, *P*=0.137) among the three groups. A retrospective power analysis was calculated and found the computed power was 0.887 in the euploidy rate analysis.

## Discussion

One of the key roles of SER is calcium storage and release, which contributes to oocyte fertilization. Moreover, complexes of endoplasmic reticulum and associated mitochondria play a crucial role in energy accumulation, protein and lipid production, and production of nuclear membranes throughout early embryo development (24).

The presence of the SER dysmorphism was considered to disturb calcium stores and oscillations, which in turn could affect fertilization and early embryo development (1). Several studied reported a significantly reduced fertilization rate in SERa+ cycles as compared to SERa− cycles (1, 25, 26). However, most studies assessing the fertilization rate after did not find any significant difference between SERa+ with SERa− cycles (10, 27, 28). Similar conflicting data were also observed when comparing SERa oocytes with SERa− oocytes. The impacts of SERa on embryo development and subsequent quality are also conflicting (29). Our data have not shown any difference in fertilization or blastocyst formation rates between SERa+ cycles and SERa– cycles. This is similar to most of the results of previous studies.

After finding that the clinical and neonatal outcomes of embryos derived from SERa+ oocytes were not impaired in our recent study (13), we compared the ploidy rates of blastocysts derived from SERa+ oocytes, sibling SERa- oocytes, and MII oocytes in the SERa- cycles. To ensure the accuracy of the data, PGT-SR cycles were excluded for analysis, for patients with chromosomal structural rearrangements may decrease in embryo development and blastocyst euploidy rate. Then, we calculated blastocyst ploidy rates for women ≤ 35 years to avoid the confounding factor of maternal age. Consistent with our data of oocyte SERa on clinical pregnancy rate and live birth rate, we found the euploid rate of blastocysts derived from SERa+ oocytes and sibling SERa- oocytes are not impacted.

Most chromosome abnormalities and first trimester embryonic aneuploidy were thought to originate from female-specific error in the first meiotic division (30). Recently, Otsuki and colleagues reported that the incidence of mitotic cleavage failure and the incidence of meiotic cleavage failure during the second polar body extrusion in oocytes with SERa were significantly higher than that in oocytes without SERa. Based on these observational results, they speculated the blastocysts originating from SERa+ oocyte may further turn into aneuploidy (2). However, direct visualization of meiotic spindle suggested the organization of the meiotic spindle is not affected by SERa (31). Through direct NGS analysis of blastocyst trophectoderm ploidy in selected PGT cycles, our result demonstrated for the first time that euploid rates of blastocysts derived from SERa+ cycles and oocytes are not impaired.

Our results showed that SERa+ cycles and oocytes had no adverse effects on fertilization, embryo development, euploid rate, and clinical outcome. However, the long-term effects, such as whether the epigenetic changes exist in SERa+ embryos, are still unknown. Since possibly more than 80% of the IVF centers transfer SERa+ embryos, more data of the birth outcome derived from SERa+ cycles and oocytes are needed.

Some limitations of our research should be noted. The nature of SERa makes it a retrospective study, which by nature cannot exclude heterogeneity. Furthermore, three forms of SERa can be classified by size (3); a bias might have been introduced because we can only observe the large and medium SERa under light microscopy. Moreover, fewer blastocysts derived from SERa+ oocytes were included for ploidy analysis after excluding women with advanced age. The results should be interpreted with caution.

## Conclusion

The euploidy rates of blastocysts derived from SERa+ cycles and oocytes may not be impacted.

## Data Availability Statement

The data that support the findings of this study have been deposited into CNGB Sequence Archive (CNSA) of China National GeneBank DataBase (CNGBdb) with accession number CNP0003208.

## Ethics Statement

Ethics Committee of Guangzhou Women and Children’s Hospital has approved the study. Written informed consent for participation was not required for this study in accordance with the national legislation and the institutional requirements.

## Author Contributions

LS was responsible for the conception and design of the study; interpretation of data; revised the article critically for important intellectual content; and approved the final draft for publication. JX and LY contributed to collect the data, analysis and interpretation of data; draft and revise the whole article. Z-HC, M-NY, and JC contributed to collecting the data, drafting and revising the article for important intellectual content. All authors contributed to the article and approved the submitted version.

## Conflict of Interest

The authors declare that the research was conducted in the absence of any commercial or financial relationships that could be construed as a potential conflict of interest.

## Publisher’s Note

All claims expressed in this article are solely those of the authors and do not necessarily represent those of their affiliated organizations, or those of the publisher, the editors and the reviewers. Any product that may be evaluated in this article, or claim that may be made by its manufacturer, is not guaranteed or endorsed by the publisher.
